# A Retrospective Analysis of Agricultural Herbicides in Surface Water Reveals Risk Plausibility for Declines in Submerged Aquatic Vegetation

**DOI:** 10.3390/toxics5030021

**Published:** 2017-09-06

**Authors:** Kelly W. Powell, W. Gregory Cope, Catherine E. LePrevost, Tom Augspurger, Annette M. McCarthy, Damian Shea

**Affiliations:** 1 Department of Applied Ecology, North Carolina State University, Raleigh, NC 27695-7617, USA; kwp1121@aol.com (K.W.P.); greg_cope@ncsu.edu (W.G.C.); 2 U.S. Fish and Wildlife Service, Ecological Services Field Office, Raleigh, NC 27636-3726, USA; tom_augspurger@fws.gov; 3 Food and Drug Administration, Office of Food Additive Safety, Center for Food Safety and Applied Nutrition, College Park, MD 20740, USA; annette.mccarthy@fda.hhs.gov; 4 North Carolina State University, Department of Biological Sciences, Raleigh, NC 27695-7633, USA; d_shea@ncsu.edu

**Keywords:** herbicides, pesticides, SAV, estuary, decline

## Abstract

The Albemarle-Pamlico Estuarine System (APES) is the second largest estuarine system within the mainland of the United States and is estimated to have lost about half of its submerged aquatic vegetation (SAV) over the past several decades. The issue of herbicide runoff and subsequent toxic effects to SAV is important because of the extensive agricultural production that occurs in the APES region. The aim of this study was to conduct a retrospective analysis of herbicide influx to waters of the APES region during the time period of documented SAV declines and to compare the measured concentrations to SAV toxicity thresholds and changes in agricultural land use. Surface water grab samples were collected at 26 sites in the APES region during May through July 2000. The most consistently measured herbicides were alachlor, atrazine, and metolachlor with geometric mean concentrations ranging from 29 to 2463 ng/L for alachlor, 14 to 7171 ng/L for atrazine, and 17 to 5866 ng/L for metolachlor. Concentrations of alachlor, atrazine, and metolachlor measured in water samples from the APES region in 2000 exceeded several of the established benchmarks, standards, or guidelines for protection of aquatic plants. Although this evaluation was of point-in-time herbicide samples (year 2000) and not analyzed for all possible herbicides used at the time, they were taken during the period of SAV declines, reveal the plausibility of exposure risk to SAV, and suggest that herbicide runoff should be studied along with other variables that influence SAV growth and distribution in future studies.

## 1. Introduction

The Albemarle and Pamlico Sounds are the two primary water bodies that comprise the Albemarle-Pamlico Estuarine System (APES). The APES is the second largest estuarine system within the mainland of the United States (U.S.). This system includes over 3000 square miles of open water and encompasses eight major sounds: Albemarle, Pamlico, Back, Bogue, Core, Croatan, Currituck, and Roanoke. Forty-three counties in North Carolina and 38 counties in Virginia drain water into the APES, creating a 31,478 square mile Albemarle-Pamlico watershed. This watershed is, in turn, emptied by multiple major river systems including the Chowan, Neuse, Pasquotank, Roanoke, Tar-Pamlico, and White Oak. The APES ecosystem contains 9299 miles of freshwater rivers and streams [1]. In general, many freshwater rivers discharge into the western side of the system while a chain of barrier islands on the eastern boundary barricade the system from the Atlantic Ocean, except for a few inlets that join the Atlantic Ocean and the southern area of the Pamlico Sound [2]. The APES area is populated by about 3.9 million people [3] and contains 4.8 million acres of agriculture land, encompassing 16,000 farms [4,5].

A vital feature of the APES is its submerged aquatic vegetation (SAV), which factors into the standing of a healthy estuary system [3]. With the exception of Florida, North Carolina has the most extensive acreage of SAV of any state on the Atlantic coast, with approximately 99% of SAV found in the Albemarle-Pamlico Estuary. An aerial survey of the Albemarle-Pamlico Estuary conducted in 2007 by the Albemarle-Pamlico National Estuary Partnership (APNEP) documented 138,741 acres of SAV [6]. The North Carolina Division of Marine Fisheries (NCDMF) estimated that there exists between 134,000 to 200,000 acres of SAV along the coasts of North Carolina [7]. Although not precisely measured and based on subjective reports in and around 1997, North Carolina, including the APES, is deemed to have lost about half of its SAV, particularly on the mainland side of coastal sounds [8,9]. A 1991 report documented that SAV had nearly vanished from the Pamlico River and Back Bay regions, that the Currituck Sound had a reduction in the density and acreage of SAV along with a change in species composition, and that a deterioration of SAV was seen in the western area of the Albemarle Sound. The report indicated, however, that the eastern expanses of the Albemarle and Pamlico Sounds exhibited no state of SAV decline [2]. Taken together, these reports indicate declines in SAV over the past several decades.

There are many potential factors contributing to the observed declines in SAV, such as water clarity (including light penetration into the water column), water depth, sediment composition, water currents, salinity, the presence of plankton and toxic blooms, invasive species, nutrient enrichment, dissolved oxygen levels, and herbicides [10,11]. Among the many threats to SAV, the issues of herbicide runoff, exposure, and toxic effects are important because of the extensive agricultural production that occurs in estuarine drainage areas.

Although now dated, but contemporary to when samples were collected in this evaluation, a 1992 National Oceanic and Atmospheric Administration (NOAA) report of agricultural land and pesticide use in the U.S., including the APES region, stated that there were approximately 282 million acres of harvested cropland, with about 31.8 million acres of that land in the nation’s estuarine drainage areas. The NOAA report estimated that 29.4 million pounds of the 35 pesticides inventoried were applied in 1987 in these estuarine drainage areas, including almost 4.0 million pounds, the second highest national use, applied in the APES area. NOAA reported that herbicides accounted for 85 percent of national pesticide use with more than 6.5 million pounds applied in the South Atlantic. Of this national use percentage for herbicides, alachlor and atrazine encompassed 45 percent or more than 9.0 million pounds being applied in national estuarine regions [12].

In 1999, the APES incorporated approximately 2.5 million acres of farmland [13], or 30 percent of its land use, which included soybean and cotton crops [14]. About 6.0 million pounds of pesticides were applied that year from March through September [15]. In North Carolina, the counties of Beaufort and Hyde on the western side of the Pamlico Sound were prototypical of the APES region, in which the primary source of agricultural income was generated from production of cotton, corn, and soybean crops.

The aim of this project was to evaluate the potential role of agricultural herbicides in the decline of SAV. The specific objectives were to assess herbicide concentrations measured in surface waters of the APES collected near the time of documented SAV declines, which has been detailed as far back as 1991 [2] and as recent as the 2010s [8,10,11]; to evaluate the risk thresholds for SAV (non-target aquatic plants) with current aquatic life criteria and standards; to compare these data to current agricultural practices and pesticide loadings; and to identify potential research and management needs for conserving SAV. This evaluation illustrates the value of historic data and the perspective that they can provide in an alternative context, that is, their recently understood potential relevance to the declines of SAV that were occurring and have continued to occur in this area since the original study [15] was conducted.

## 2. Materials and Methods

### 2.1. Sample Collection and Analysis

**Figure 1:**
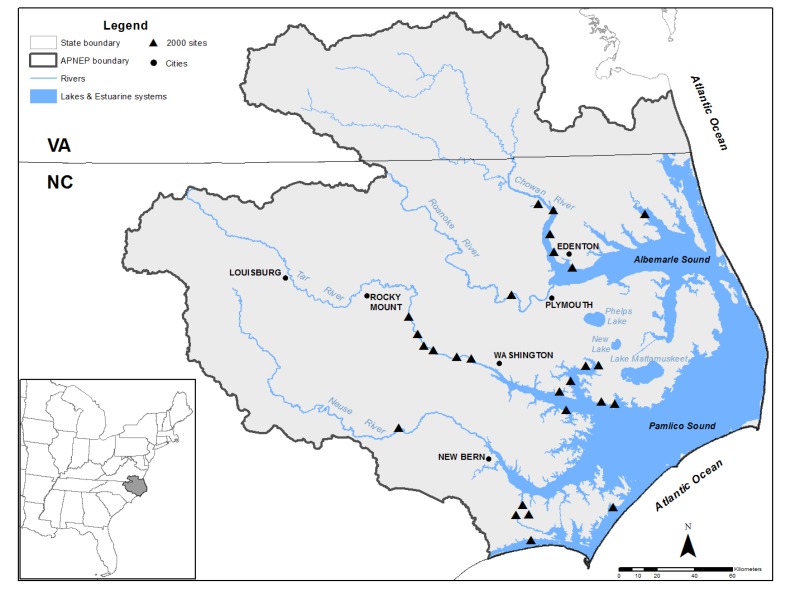
Map of the 26 sites in the Albemarle-Pamlico Estuarine System where surface water samples were collected in 2000 for herbicide analysis.

Surface water grab samples were collected monthly at 26 sites from seven riverine and estuarine locations in the APES region from May through July 2000 (Figure 1).

All samples were taken according to North Carolina Department of Environment and Natural Resources (NCDENR) protocols. Briefly, water was collected with a point-sample grab in Teflon-lined amber jars from a location in the center of the river channel at each site. Once collected, the samples were stored on ice in a cooler and transported to the Analytical Toxicology Laboratory at North Carolina State University for processing and analysis. At the laboratory, samples were stored refrigerated at 4 °C and extracted within 24–48 h of collection. All water samples were first filtered in the lab with glass microfiber filters (Whatman, Maidstone, UK, GF/B, 1 μm pore size) and then nylon filters (Osmonics Inc., Macungie, PA, USA, 0.45 μm pore size). The filtrates were extracted using solid phase extraction with Empore C-18 Extraction disks (3M-Corporation, Maplewood, MN, USA, 47 mm diam.). For these samples, 22 herbicides, 14 insecticides, 1 fungicide, and 3 herbicide metabolites were analyzed [15]. Only the herbicide and herbicide metabolite data are presented and evaluated in this paper because of the relevance to SAV. A second set of water samples was collected bi-weekly from March through October 2000 from drainage ditches located on a large commercial farm in Beaufort County, North Carolina, to assess potential herbicide transport and measurement in relation to timing of application. For these samples, analysis focused on the primary pre-emergent herbicide (atrazine) and post-emergent herbicide (cyanazine) used in the area, along with their primary metabolites (atrazine-desethyl and cyanamide-amide, respectively). These water samples were processed similarly to those described above, except that extraction was performed with mixed-mode extraction cartridges to achieve better recovery of the metabolites (Oasis HLB, Waters Corp., Milford, MA, USA).

At the time of processing, sample volumes were measured and spiked with three surrogate internal standards (diazinon-d_10_, alpha-HCH-d_6_, turbuthylazine) prior to extraction. C-18 Empore Extraction disks were conditioned using ethyl acetate and methanol. Samples were extracted using the Empore C_18_ extraction method described by Mueller et al. [16] or using the Oasis HLB cartridge method described by Chaves et al. [17] and extracts were concentrated under N_2_ gas and filtered through a 0.45 µm PTFE filter prior to analysis. All samples were analyzed by GC-MS (Agilent Model 6800 with a 5973 detector, Agilent Technologies, Santa Clara, CA, USA) with the method described by Zuagg et al. [18], which was modified to specifically include the 22 herbicides and metabolites of interest. See McCarthy [15] for additional details on sample collection and analysis.

During analysis, a rigorous quality assurance protocol was followed. With each batch of samples analyzed (10–20 samples), there were blanks, duplicate samples, matrix spike samples, and matrix spike sample duplicates. None of the pesticides reported here were detected in any of the blanks. The relative percent difference for all duplicate samples was less than 25%, and the recovery of the three surrogate internal standards averaged 86.4% (range 79.8–96.1%). The limit of detection (LOD) for all herbicides measured was approximately 1.0 ng/L. If a herbicide concentration was not detected in a given sample, the value used for statistical procedures (i.e., calculation of geometric mean) was expressed as 50% of the LOD.

### 2.2. Sources for Toxicity Thresholds to Aquatic Life, Including SAV, and Pesticide and Land Use

For the herbicides evaluated, we obtained all available toxicity thresholds for aquatic life and other relevant available criteria and benchmarks from the U.S. Environmental Protection Agency (US EPA), Office of Pesticide Programs (OPP) [19]; NCDENR, Division of Water Resources [20]; and the Canadian Council of Ministers of the Environment (CCME) [21]. To assess the potential impacts of the herbicides of interest on SAV, specific toxicity thresholds for SAV were taken from the US EPA, OPP Aquatic Life Benchmark document for freshwater vascular plants.

The number of farms and acres of crop production type in the APES region were obtained from the US Department of Agriculture (USDA) Census of Agriculture for 1997, 2002, 2007, and 2012 for 12 of the 13 APES counties in North Carolina; Dare County data were undisclosed because the limited number of farms in the county prevented data release due to confidentiality laws restricting release of information that would allow a user to track data to a specific farm. Agricultural land use for analysis of crop type among years was obtained through the USDA National Agricultural Statistics Service (NASS) web-based mapping application CropScape, which utilizes a Cropland Data Layer for researching and publishing geospatial cropland data. This tool was explicitly used for analysis of crop production changes in Beaufort and Hyde counties in North Carolina for 2002, 2008, 2010, and 2012. The total pounds of pesticides applied and crop-type production acreage for 1999 were compiled by McCarthy [15]. The annual sum totals of pounds of pesticide active ingredients sold in North Carolina from 2006 through 2013 were obtained through GfK Market Research [22].

## 3. Results

### 3.1. Herbicides in Surface Water

**Figure 2:**
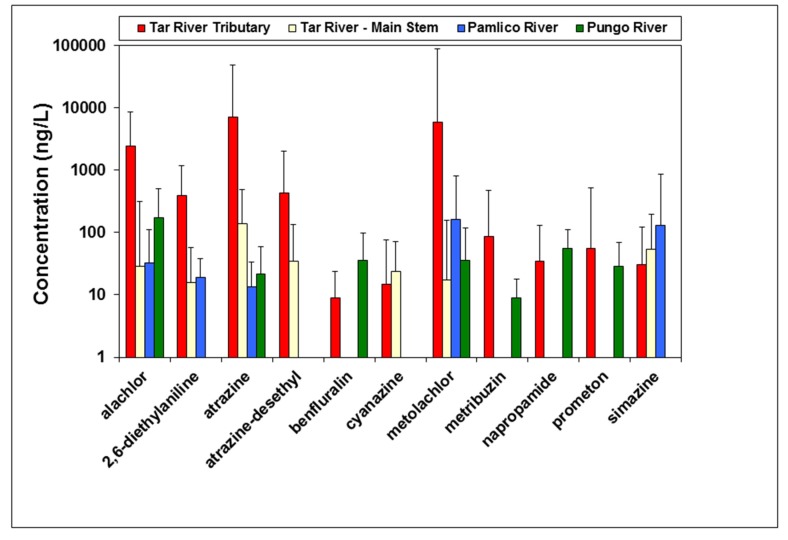
Geometric mean and maximum (denoted by top of range bar) herbicide and metabolite concentrations (ng/L) measured in samples of surface water collected in the Tar River tributaries, Tar River mainstem, Pamlico River, and Pungo River in 2000. Data are not shown where herbicide concentrations were not detected (below 1 ng/L).

A total of nine herbicides and two herbicide metabolites were consistently measured among the 26 sites in the main water bodies sampled (Figure 2) during the year 2000. For these locations, the herbicides alachlor, atrazine, and metolachlor were predominant (Figure 2). One additional herbicide (pendimethalin) not shown in Figure 2 was measured in surface water samples from the 26 sites in the APES region in 2000 (Table S1), and one additional herbicide metabolite (cyanazine-amide) was measured in surface water samples from the large commercial farm in Beaufort County.

A comparison of spatial coverage of sampling locations among the Tar, Pamlico, and Pungo River drainages showed that the greatest concentrations of these herbicides were measured in the Tar River tributaries in the freshwater portion of the river (upstream of Washington, North Carolina) (Figure 2), as compared to the brackish Pamlico and Pungo Rivers. Within each of the 26 sample sites, the geometric mean of concentrations was calculated over the period of sampling. Geometric mean concentrations for the predominant herbicides among locations ranged from 29 to 2463 ng/L for alachlor, 14 to 7171 ng/L for atrazine, and 17 to 5866 ng/L for metolachlor. The maximum concentrations of these three herbicides measured during any single sampling period were 6100 ng/L for alachlor, 41,000 ng/L for atrazine, and 82,000 ng/L for metolachlor. The maximum concentrations for these herbicides were all measured in the Tar River tributaries. The alachlor metabolite 2,6-diethylaniline and the atrazine metabolite atrazine-desethyl were the two primary herbicide breakdown products measured in surface waters from the region. The cyanazine metabolite cyanazine-amide was measured at one location. No other breakdown products were measured (i.e., quantified above detection limits).

**Figure 3:**
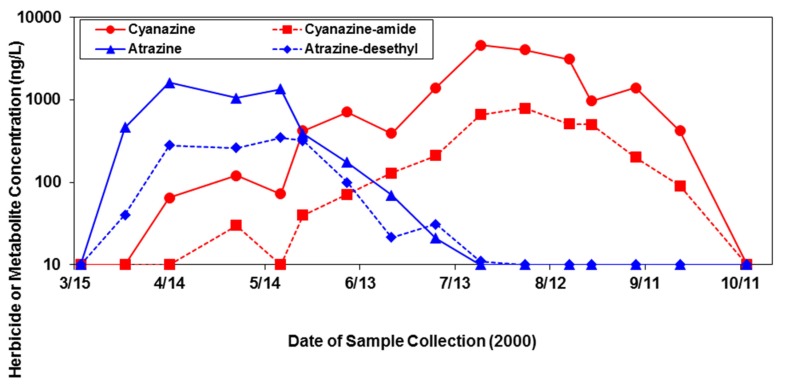
Herbicide and associated metabolite concentrations (ng/L) measured in samples of surface water collected from drainage ditches located on a large commercial farm in Beaufort County, North Carolina during the 2000 growing season.

In evaluating potential herbicide transport and measurement in relation to timing of application, the water samples taken over time from the ditches near the Beaufort County farm showed that atrazine, used as a pre-emergent herbicide on corn, demonstrated peak concentrations of 1050 to 1600 ng/L in the April–May period after its spring application around mid-March. Likewise, its main metabolite, atrazine-desethyl, exhibited a similar pattern, but in lesser concentrations (Figure 3). Concentrations of cyanazine, used as a post-emergent herbicide on corn and cotton, and its major metabolite, cyanazine-amide, peaked later in the season (July–August) than atrazine, and attained greater maximum sustained concentrations (3100–4600 ng/L for cyanazine, 510–790 ng/L for cyanazine-amide) compared to atrazine (Figure 3).

### 3.2. Toxicity Thresholds for Aquatic Life, Including SAV, and Pesticide and Land Use

Most of the herbicides evaluated in this study did not have published aquatic life benchmarks, criteria, standards, or guidelines, especially for aquatic vascular and nonvascular plants. However, we were successful in compiling from various federal and state published sources the comparative information for the three predominant herbicides (alachlor, atrazine, and metolachlor) measured in this evaluation (Table 1).

In comparing the overall measured geometric mean and maximum concentrations of these herbicides to the current criteria, we found that the predominant herbicides measured in water samples from the APES region in 2000 exceeded several of the established benchmarks, standards, or guidelines. For alachlor, the measured maximum concentration of 6100 ng/L exceeded the US EPA OPP Aquatic Life Benchmarks of 1640 ng/L and 2300 ng/L for nonvascular and vascular plants, respectively (Table 1). The measured geometric mean concentration of atrazine exceeded the US EPA OPP Aquatic Life Benchmark for nonvascular plants, and the measured maximum concentration of atrazine exceeded the US EPA OPP Aquatic Life Benchmarks for both nonvascular and vascular plants. Both the measured geometric mean concentration of atrazine and the measured maximum concentration of atrazine exceeded the CCME Water Quality Guideline of 1800 ng/L for the protection of freshwater organisms (Table 1). Likewise, the measured maximum concentration of metolachlor exceeded the CCME Water Quality Guideline for the protection of freshwater organisms.

As mentioned previously, 13 counties in North Carolina comprise the APES region, including Beaufort and Hyde. Dare County information is typically not disclosed due to the confidentiality issues related to the small number of farms in the county. Therefore, our assessment of the crops produced, changes in agricultural land use, pesticides applied, and the number of farms in the region were based on the remaining 12 counties. We found that for the years 1997 through 2012 (specifically comparing 1997, 2002, 2007, and 2012) the number of farms remained relatively constant (range 1984–2024) until 2007. From 2007 to 2012, the number of farms decreased significantly from 1984 to 258 [23], which is consistent with general trends in farm numbers nationwide. At the same time, the percent of acres harvested in the 12 counties for corn and soybeans remained steady from 1997 to 2012, averaging 35% for corn (range 32–38%) and 48.5% for soybeans (range 45–51%). In contrast, cotton averaged 16.5% with a substantial increase from 13 to 23% from 1997 to 2002.

**Figure 4:**
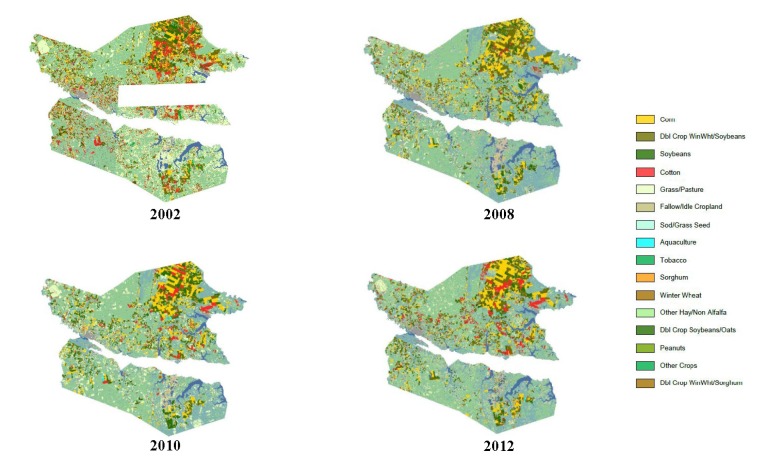
Beaufort County, NC agriculture land cover by crop category. Transparent sections contained no data (USDA NASS, CropScape, Cropland Data Layer).

**Figure 5:**
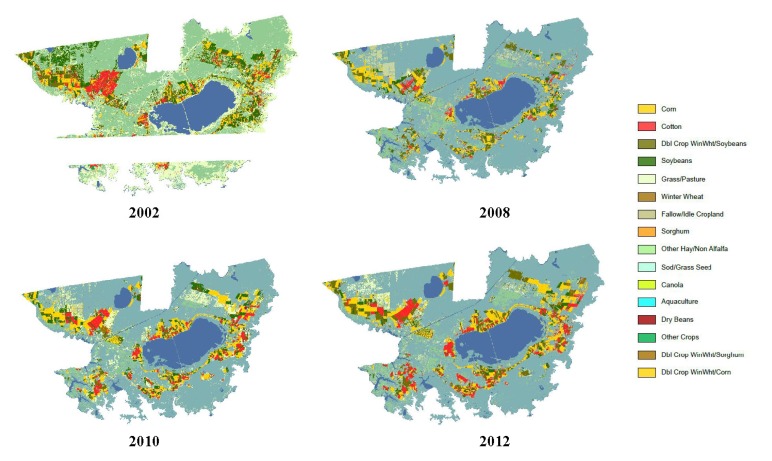
Hyde County, NC agriculture land cover by crop category. Transparent sections contained no data (USDA NASS, CropScape, Cropland Data Layer).

Specifically in Beaufort and Hyde counties, the percent of acres harvested for cotton rose sharply from 4 to 24% and from 6 to 23%, respectively, from 1997 to 2002. The percent of acres harvested for corn in these two counties remained consistent according to the agricultural census data for those years, while the percent of acres harvested for soybeans fluctuated for both counties. In 1997, the percent of acres harvested for soybeans in Hyde County was 50%. However, subsequent census years indicated an average of 37% for soybean acres harvested in this county. Our geospatial analysis from CropScape visualized these changes in crop types among years in Beaufort and Hyde counties (Figure 4 and Figure 5). These data demonstrate the changes (increases) in cotton acres for both counties from 2008 to 2012, especially in Hyde County near the Lake Mattamuskeet area (Figure 5).

Concomitantly with changes in crop type, the sum total pounds of herbicide active ingredients applied overall within the region, and within Beaufort and Hyde counties specifically, also fluctuated. For example, in 1999, the estimated use rates (pounds of active ingredient applied) of herbicides in Beaufort and Hyde counties ranged from 35,000 to 70,000 for alachlor, 34,000 to 60,000 for atrazine, and 23,000 to 40,000 for metolachlor [15]. For the period 2006 through 2009, the sum pounds of alachlor sold in North Carolina increased from 43,234 to 404,889, but has since steadily decreased to 61,248 in 2013. Atrazine ranked highest of the predominant herbicides in the sum of pounds of active ingredient sold from 2006 through 2010 (mean 1,084,889, range 720,227–1,174,829 pounds). From 2011 through 2013, the pounds of atrazine sold decreased to an average of 896,633 (range 696,140–1,025,747), and during this most recent time period, only more pounds of *S*-metolachlor were sold (mean 1,176,469). Of the herbicides evaluated, the lowest sum of pounds sold from 2006 through 2013 was for metolachlor (range 1672–61,455).

## 4. Discussion

Our retrospective analysis of the herbicides and metabolites measured in surface waters of the APES region in the year 2000 were dominated by three compounds: alachlor, atrazine, and metolachlor. These results resonate with previous sampling efforts of the Tar-Pamlico River Basin, in which annual samples taken from 1992 to 2001 identified atrazine in 38% to 92% of the samples and metolachlor in 73 to 100% of the samples [10]. The greatest concentrations of herbicides were measured in the Tar River and its tributaries (Figure 2). The Tar River is a 346 km long, southeast flowing freshwater river that transitions into the brackish Pamlico River near the town of Washington, North Carolina (Figure 1). Therefore, the freshwater portion of the river contained the greatest measured concentrations of these herbicides.

In comparing the APES region to other estuarine systems in the U.S., the Chesapeake Bay system has had similar periodic monitoring of pesticide influx. In 2000, samples of surface water were taken at 18 stations along the Chesapeake Bay mainstem and analyzed for a suite of pesticides [24]. Similar to our 2000 findings, atrazine and metolachlor were measured at all stations in their study. Another study of the Chesapeake Bay system was conducted in 2004 (also reported in [24]) during which 61 stations within five tidal areas were analyzed for pesticides in surface water. That study measured atrazine in all samples from the five tidal areas and specifically identified the Chester River as having metolachlor in 100% of samples; this river also contained the maximum measured concentration of atrazine (820 ng/L). The McConnell et al. [24] study did not measure concentrations of alachlor. Among the 26 sampling sites in the APES in 2000, again, the maximum concentrations of atrazine and metolachlor were 41,000 ng/L and 82,000 ng/L, respectively. By comparison, in McConnell et al. [24], the maximum concentration of atrazine was 820 ng/L as recorded in 2004 in the Chester River, while the maximum for metolachlor ethane sulfonic acid (MESA) was documented at 2900 ng/L in the Nanticoke River.

Another study in the Chesapeake Bay system conducted in 1996 showed that for Patuxent River Estuary water samples, atrazine was measured at all stations and, overall, was found in the highest concentrations (1290 ng/L) of all the target compounds [25]. While metolachlor was detected in their study at two upstream locations, the concentrations were typically below the limit of detection at downstream sites. Alachlor was found only at two upper river locations [25].

For comparison, along the west coast of the U.S. in July of 1999 and 2000, the San Francisco Estuary Regional Monitoring Program (RMP) collected water samples during a study directed at the identification of previously unmonitored synthetic organic contaminants in the San Francisco Estuary. The pesticides detected included atrazine (81 ng/L) and metolachlor (89 ng/L), both of which were found in the Delta region, which is made up of the Sacramento and San Joaquin Rivers. Metolachlor was also measured at a concentration of 35 ng/L in the North Bay, which included the Petaluma River, San Pablo Bay, Napa River, and Grizzly Bay [26].

In and around the year 2000, only the data evaluated here and the few studies just mentioned had measured the extent of pesticide contamination in the nation’s estuaries. Prior to this time, the majority of studies in U.S. estuarine systems were largely focused on organochlorine pesticides. In the APES region, prior to the analyses of McCarthy [15], few studies had been performed to evaluate pesticide contamination; those that had been done were concentrated on the region known as the inner coastal plain of the APES [27]. The pesticide detection rates in the inner coastal plain from those studies were then among the highest nationally [28,29].

The congruent findings of our study and the other studies previously discussed from estuary systems around the U.S. during this time were supported by a USGS Scientific Investigations Report that summarized pesticide use trends in the U.S. from 1992 through 2010. It was found that by 2007 more than 1131 million pounds of pesticides were used for agricultural and nonagricultural uses, with periods of increase and decrease in agricultural uses throughout, but that the pesticides most commonly used in agricultural applications were the herbicides alachlor, atrazine, and metolachlor [30].

Concentrations of alachlor, atrazine, and metolachlor measured in surface water samples from the APES region in 2000 exceeded several of the established benchmarks, standards, or guidelines for protection of aquatic life. The measured concentrations of these chemicals did not, however, exceed benchmarks for fish and invertebrates. The presence of these compounds along with the exceedance of these benchmarks, especially the US EPA OPP Aquatic Life Benchmark of nonvascular and vascular plants for alachlor and atrazine, supports the plausibility for certain agriculturally applied herbicides to runoff and elicit toxic effects on SAV.

Moreover, our findings relating herbicide transport and measurement in surface waters to the timing of application (Figure 3), demonstrate that herbicides are present in the water at relatively high concentrations at a time period (i.e., early spring) when young or immature SAV is actively growing and susceptibility to herbicide exposures may be greater (or different) than in mature plants.

For example, experimentally observed physiological effects of herbicides, including atrazine, on SAV include decreased relative growth rate and chlorophyll-*a* content, and differences in sensitivity to herbicides have been noted among SAV species [31]. Specifically, atrazine has been found to affect SAV root growth and node development [32]. Because its mechanism of action depends on light, however, atrazine is thought to have diminished toxicity in surface waters experiencing turbid conditions, as might likely be the case in waters receiving agricultural runoff [33]. Previous studies have suggested that atrazine does not cause considerable risk to aquatic plants, with established EC_50_ values for growth and photosynthesis at 22 to 474 μg/L for SAV and algae; however, fast-growing SAV tends to be more sensitive to atrazine than slow-growing plants [34]. Also, as shown by our analysis, SAV can be exposed to multiple herbicides simultaneously. If a particular SAV species were sensitive to multiple herbicides present in the system, such as a class of herbicides like the triazines, which represented two of three predominant herbicides measured during collection of our data, the outcome could be detrimental to that species when considering combined or cumulative toxicities [34].

Herbicides have been previously implicated in the decline of SAV [35]. Aquatic plants have a relatively large exposure surface area and duration, particularly in stagnant or minimally flowing waters, and thus, their sensitivity to herbicides may be greater than those of certain terrestrial plants. Conversely, diluted herbicides in surface water runoff could be less toxic to aquatic plants than to terrestrial species [36], although in tidally influenced waters, like many of those in the APES region, the transport and movement of herbicides with tidal action [37] may result in SAV being repeatedly exposed with pulses of herbicides.

In addition to herbicide toxicity, SAV decline in the APES and elsewhere may be attributed to other factors, including decreased light penetration due to sedimentation and excess phytoplankton growth related to nutrient enrichment [8,10,11]. While herbicides in surface waters may not affect healthy plants or animals, the presence of herbicides may adversely affect sensitive species already experiencing stressful conditions such as habitat degradation or poor water quality [38]. Dantin et al. [39] assessed the toxic effects of chemical stressors, including herbicides, on aquatic plants in the presence of excessive nutrients. Under experimental settings, the field-collected species of SAV were pre-treated to three nutrients (ammonium, nitrogen, and phosphorous) for 3 months and then exposed to 11 and 110 μg/L of atrazine for 96 h. By measuring the chlorophyll fluorescence activity, they found that nutrient pre-treatment increased the toxicity of atrazine. Even in the presence of low and ambient nutrient concentrations and average levels of atrazine, the electron transport rates were 48 to 59% less. They concluded that the presence of nutrients should be considered in risk assessments, especially in those involving phytotoxicants and non-target vascular plants [39]. Other variables implicated in SAV declines include diminished light penetration from harmful algal blooms and invasive plant species that shade or outcompete native species of SAV [10], as well as rising sea levels that have led to increased salinity (saltwater intrusion) in certain areas and its associated shoreline transformation and increased depth resulting in unsuitable habitat [40,41,42].

Our assessment of the crops produced, changes in agricultural land use, herbicides applied, and the number of farms in the APES region showed that, although there were expected fluctuations, the percent of acres harvested in the 12 counties for corn and soybeans remained steady from 1997 to 2012, averaging 35% for corn and 48.5% for soybeans. In contrast, cotton averaged 16.5% with a significant increase from 13 to 23% from 1997 to 2002. For corn crops, alachlor and atrazine were typically used. Alachlor was also used for soybean and peanut crops, and the pre-emergent chloracetanilide herbicide metolachlor was regularly used on corn, cotton, and sorghum [12,15]. Thus, changes in agricultural land use (i.e., specific crops grown), associated herbicide use on those crops, and measurements of herbicides in adjacent surface waters sometimes exceeding safety thresholds or guidelines are multiple lines of evidence of potential herbicide exposure and effects on SAV that were observed during this time period. In Beaufort and Hyde counties, where the percent of acres harvested for cotton rose sharply from 4 to 24% and from 6 to 23%, respectively, from 1997 to 2002 (Figure 4 and Figure 5), there would have been a concomitant increase in the use cotton herbicides (and potentially growth regulators and defoliants used in cotton production), which may have adversely affected SAV in the adjacent waters and specifically in Lake Mattamuskeet where few herbicide samples have been taken and analyzed [11].

## 5. Conclusions

Native SAV provides critical habitat (food and cover) for many waterfowl and fish species, and its loss may be influencing their population density, diversity, and distribution, as well as overall water quality through diminished ecosystem services [11,42]. Many factors are known to influence the presence of SAV, and agricultural herbicide runoff and toxicity is among them. The potential adverse effects of herbicides have been a concern over the extended time period of SAV losses in the APES region, but targeted, in-depth studies addressing the topic have not been done.

Although this evaluation was of point-in-time (year 2000) herbicide samples, which were not analyzed for all possible herbicides used at the time (e.g., glyphosate on genetically modified corn and soybeans), they were taken during the period of SAV declines, revealed the presence of potentially toxic concentrations in surface waters, and infer the plausibility of exposure risk to SAV. Moreover, new herbicide products may have been introduced to the market and used during this period of continuing declines in SAV in waters of the region, since the time our samples were analyzed. Therefore, we suggest that herbicide runoff should not be discounted in the historic and ongoing declines of SAV and should be studied along with other variables that influence SAV growth and distribution in future studies. Specifically, we recommend that targeted, hypothesis-driven laboratory and field research and monitoring are needed to further assess the relations between herbicide exposures and SAV decline in the APES, including in Lake Mattamuskeet, by incorporating the potential impacts of varying SAV species sensitivity, cumulative effects of herbicide mixtures, and influence of pre-existing natural or anthropogenic stressors such as climate change and nutrient enrichment.

## Figures and Tables

**Table 1:** Comparison of mean and maximum measured concentrations (ng/L) of alachlor, atrazine, and metolachlor measured at 26 sites in the Albemarle-Pamlico Estuarine System and their corresponding aquatic life protection benchmarks, standards, and guidelines from the U.S. Environmental Protection Agency (US EPA), Office of Pesticide Programs (OPP) (EPA 2014); NCDENR, Division of Water Resources (NCDENR 2013); and the Canadian Council of Ministers of the Environment (CCME; CCME 2014).

Herbicide	Measured Geometric Mean Concentration	Measured Maximum Concentration	US EPA OPP Aquatic Life Benchmarks		NC Surface Water Aquatic Life Standards		CCME Water Quality Guidelines ^a^
			Nonvascular Plants	Vascular Plants	Freshwater	Saltwater	Freshwater
Alachlor	674	6100	1640	2300	NA	NA	NA
Atrazine	1836	41,000	1000	37,000	150,000	76,000	1800
Metolachlor	1521	82,000	NA	NA	240,000	NA	7800

NA, data not available; ^a^ Marine CCME Water Quality Guidelines do not exist for displayed compounds.

## Supplementary Materials

The following are available online at www.mdpi.com/2305-6304/5/3/21/s1, Table S1, Herbicide concentrations (ug/L) measured in water samples collected from the 26 sites in the Albemarle-Pamlico Estuary System in 2000.
